# Chikungunya Virus or Chlamydia pneumoniae: Attributing a Post-infectious Arthropathy on a Single Immunoglobulin G in Klebsiella pneumoniae Bacteremia

**DOI:** 10.7759/cureus.117505

**Published:** 2026-10-05

**Authors:** Mukudzeishe T Zibanayi, Rio G Connell, John H Frederick

**Affiliations:** 1 Surgery, Ross University School of Medicine, Bridgetown, BRB; 2 Surgery, St. George's University, St. George, GRD; 3 Hospital Medicine/Internal Medicine, AdventHealth Orlando, Orlando, USA

**Keywords:** arboviral serology, chikungunya virus, diagnostic reasoning, immunoglobulin g interpretation, klebsiella pneumoniae bacteremia, polyarthralgia, post-chikungunya chronic arthritis, reactive arthritis

## Abstract

A man in his 70s from a chikungunya virus-endemic region of the Caribbean presented to a community hospital with one week of low back pain and bilateral foot pain and swelling. He was diagnosed with extended-spectrum beta-lactamase-producing *Klebsiella pneumoniae* bacteremia from a urinary source and treated with meropenem. The foot pain and swelling, documented as present on admission, were incompletely explained by the bacteremia or by the limited unilateral arterial disease found on duplex ultrasonography; a lumbar radicular contribution was raised on the day of admission and was never formally evaluated. Arboviral serology sent on hospital day 11 returned on hospital day 15 - the first day of recurrent fevers without an identified focus - as chikungunya virus immunoglobulin G positive with immunoglobulin M negative; a polymerase chain reaction obtained outside the viremic window was not detected. *Chlamydia pneumoniae* immunoglobulin G was also positive. The result was entered on the problem list as an active chikungunya infection and remained there for 11 days while a fever-of-unknown-origin workup proceeded. With non-steroidal anti-inflammatory drugs contraindicated by chronic kidney disease and acetaminophen withdrawn for cholestatic liver injury, corticosteroids were started for a presumed inflammatory arthropathy, and doxycycline was given as an inpatient course. Two attributions were then documented: the hospitalist who took over care in the fourth week recorded a probable post-chikungunya arthropathy; the discharge summary attributed it to prior *Chlamydia pneumoniae* infection. Neither attribution is established. Both rest on a single untitered immunoglobulin G that is near-universal in this patient's demographic and cannot date an infection or link it to the presenting complaint. This case is presented as an exercise in diagnostic reasoning when serology is indeterminate. The two positive immunoglobulin G results were read three ways in one admission - the chikungunya result as an active infection on the problem list, the Chlamydia pneumoniae result as an indication for antibiotics, and finally the chikungunya result as prior infection behind a post-infectious polyarthritis - and only the last reading fits the serology. The report weighs what the panel could and could not contribute and argues that a positive immunoglobulin G in this population should be read as a framing tool rather than a diagnosis.

## Introduction

Chikungunya virus is a mosquito-borne alphavirus transmitted by *Aedes aegypti* and *Aedes albopictus*, endemic to the Caribbean, sub-Saharan Africa, and South and Southeast Asia. Following outbreak years, seroprevalence in affected populations may reach 50% or more [1-3]. Post-chikungunya chronic arthritis, a symmetric inflammatory polyarthralgia clinically indistinguishable from seronegative rheumatoid arthritis, develops in approximately 25 to 50% of infected individuals depending on cohort definition and duration of follow-up and may persist for months to years after acute infection [4-6]. Persistent viral antigen and chronic inflammation have been demonstrated in affected tissue despite a robust host immune response [7].

The serologic profile of immunoglobulin G positive with immunoglobulin M and polymerase chain reaction negative identifies prior rather than active infection: immunoglobulin G persists for years to decades, while immunoglobulin M and viral RNA are confined to the acute phase [1,2]. That principle is well established. What is less well handled in practice is the situation this report describes: an inpatient with an unrelated primary diagnosis, an unexplained musculoskeletal complaint, and two post-infectious candidates, chikungunya virus and *Chlamydia pneumoniae,* each supported by nothing more than a single positive immunoglobulin G, in a patient whose age and region of origin made both results probable before the blood was drawn.

The report is therefore not about a diagnosis made. It is about reasoning when testing is indeterminate, about what serology can and cannot carry, about what happens to a predictable positive result once it is in the chart, and about constructing a treatment plan when a thorough workup ends without a definitive diagnosis. A PubMed search of the case report literature combining chikungunya terms with diagnostic-reasoning terms - ("chikungunya" OR "CHIKV") AND ("diagnostic reasoning" OR "clinical reasoning"), limited to case reports - returned no results when performed on August 27, 2026, and again on September 29, 2026.

## Case presentation

Sources of this report

The patient was cared for by several physicians over a four-week admission. One author (JHF), a hospitalist, took over his care on hospital day 21 and wrote the progress notes from then until hospital day 26; the attribution of the arthropathy quoted below is his and is taken from those notes. The other physicians involved in the patient's care, including the attending physician of record, who wrote the daily notes for the first three weeks and the discharge summary, were consulted in preparing this report. The two student authors observed the case and took no part in clinical decisions. The account is drawn from the medical record, with the source of each finding stated where it matters; where the record contains no rationale for a decision, that is stated rather than reconstructed from recollection.

History and examination

A man in his 70s presented to a community hospital emergency department in the southeastern United States with a one-week history of low back pain and progressive bilateral foot swelling and pain, with difficulty walking because of the pain. He was from a chikungunya virus-endemic Caribbean region, where his chronic kidney disease had been followed by a nephrologist; the dates of his move to the United States and of his last travel to the region were not documented. His past medical history included hypertension, coronary artery disease, chronic kidney disease, and a prior orthopedic procedure on the left lower extremity, the nature of which was not recorded. Home medications included gabapentin 300 mg twice daily, for which the indication was not documented. He denied fever, dysuria, nausea, vomiting, and abdominal pain before admission. His adult daughter, present throughout the hospitalization, supplemented the history.

Admission vital signs showed sinus tachycardia (118 beats per minute), hypertension (188/88 mmHg), a temperature of 37.1°C, and oxygen saturation of 97% on room air. Weight was 104 kg (body mass index 34 kg/m^2^).

No structured joint examination was performed at any point in the hospitalization. The emergency department template examination recorded the extremities as normal without edema; the admitting physician's history and physical examination, recorded the same afternoon, documented bilateral swelling and tenderness of the feet that did not extend up the legs, with the explicit note that the tenderness was "not focused over any particular joint" and a query of neuropathy. The vascular surgery consultation on hospital day two recorded warm, well-perfused feet with mild bilateral edema, no open wounds or discoloration, soft compartments, intact and symmetric motor and sensory function, palpable femoral, popliteal and dorsalis pedis pulses bilaterally, a palpable left and audible right posterior tibial signal, and capillary refill under two seconds. On history to the vascular surgeon, the patient localized the onset of pain to the lower back with radiation into both shins and feet, denied pain at rest, denied claudication, and described pain provoked by standing from sitting and continuing with walking; the review of systems was positive for back pain, paresthesias, and weakness. Nursing documentation on hospital day seven recorded 2+ pitting edema over the dorsum of both feet with non-pitting legs and a swollen left hand, the right hand being described as improved from the preceding day. Daily hospitalist examinations from hospital day two to discharge recorded bilateral lower-leg edema with normal range of motion and nothing further.

Investigations

Admission laboratory values are presented in Table 1: leukocytosis (white blood cell count 13.51 × 10⁹/L) with neutrophilia (lymphocytes 9.3%, reference range 21.0-45.0%; 1.25 × 10⁹/L, reference range 1.00-4.90 × 10⁹/L), normocytic anemia (hemoglobin 11.6 g/dL, mean corpuscular volume 82.5 fL), acute kidney injury on chronic kidney disease (serum creatinine 2.10 mg/dL, blood urea nitrogen 39 mg/dL, estimated glomerular filtration rate 33.2 mL/min/1.73 m²), mild transaminase elevation (aspartate aminotransferase 61 U/L, alanine aminotransferase 73 U/L), a raised globulin fraction (5.1 g/dL) with normal albumin (3.50 g/dL, reference range 3.50-5.20 g/dL), and a urinalysis with 3+ leukocyte esterase, 58 white cells per high-power field and 4+ bacteria without proteinuria. N-terminal pro-B-type natriuretic peptide (180.7 pg/mL) and lactate (1.70 mmol/L) were normal; hemoglobin A1c was 6.3%. Computed tomography of the abdomen and pelvis without contrast showed a distended bladder and perinephric stranding without hydronephrosis or calculus.

**Table 1:** Laboratory investigations on admission Reference ranges are those of the performing laboratory. eGFR: estimated glomerular filtration rate.

Parameters	Patient values	Reference range
White blood cell count	13.51 × 10^9^/L	3.30-10.60 × 10^9^/L
Hemoglobin	11.6 g/dL	13.2-17.8 g/dL
Mean corpuscular volume	82.5 fL	80.0-99.0 fL
Serum creatinine	2.10 mg/dL	0.40-1.40 mg/dL
Estimated glomerular filtration rate	33.2 mL/min/1.73 m^2^	≥60 mL/min/1.73 m^2^ (acceptable)
Blood urea nitrogen	39 mg/dL	6.0-20.0 mg/dL
Aspartate aminotransferase	61 U/L	15-41 U/L
Alanine aminotransferase	73 U/L	6-48 U/L
Urinalysis, leukocyte esterase	3+	Negative
Urine microscopy	Heavy bacteriuria	No bacteria seen

Two blood-culture sets drawn in the emergency department showed no growth. One of the two sets drawn on hospital day two grew *Klebsiella pneumoniae* with an extended-spectrum beta-lactamase phenotype; urine culture grew the same organism, establishing *Klebsiella pneumoniae* bacteremia from a urinary source as the primary diagnosis. The full susceptibility panel was not available in the records reviewed for this report. Repeat blood cultures on hospital day five and surveillance cultures on hospital day 24 showed no growth (Table 2).

**Table 2:** Microbiologic, serologic, and molecular investigations CHIKV: chikungunya virus; ESBL: extended-spectrum beta-lactamase; PCR: polymerase chain reaction; QuantiFERON-TB Gold: interferon-gamma release assay for *Mycobacterium tuberculosis*.

Parameters	Patient values	Reference range
Blood culture (admission, two sets)	No growth	No growth
Blood culture (hospital day two)	*Klebsiella pneumoniae*, ESBL phenotype	No growth
Urine culture	*Klebsiella pneumoniae*, ESBL phenotype	No growth
Surveillance blood culture (hospital day 24)	No growth	No growth
CHIKV immunoglobulin G (collected hospital day 11, results reported on hospital day 15)	3.77 (positive)	≤0.79 negative; 0.80-1.09 equivocal; ≥1.10 positive
CHIKV immunoglobulin M (collected hospital day 11, results reported on hospital day 15)	0.30 (negative)	≤0.79 negative; 0.80-1.09 equivocal; ≥1.10 positive
CHIKV PCR, venous blood (collected hospital day 16, results reported on hospital day 21)	Not detected	Not detected
*Chlamydia pneumoniae* immunoglobulin G	Positive	Negative
Zika virus serology	Negative	Negative
Dengue virus serology	Negative	Negative
Malaria blood smear	Negative	Negative
Malaria antigen	Negative	Negative
QuantiFERON-TB Gold	Negative	Negative

Bilateral lower extremity arterial duplex on admission demonstrated only trace flow in the right posterior tibial artery, which the interpreting radiologist reported as possibly indicating near-occlusive stenosis; this segment is shown in Figure 1A. The anterior tibial, peroneal, and dorsalis pedis arteries were patent with triphasic waveforms, and the patent peroneal artery is shown for comparison in Figure 1B. The left lower extremity showed no evidence of significant arterial occlusive disease. Venous Doppler ultrasound of the right upper extremity on hospital day three and of the bilateral lower extremities on hospital day 10 excluded deep venous thrombosis (Figure 2). A left upper extremity venous study was ordered on the day the hand swelling was recorded; its result was not located in the records reviewed. A transthoracic echocardiogram was ordered on hospital day four for persistent tachycardia; its result was likewise not located.

**Figure 1:**
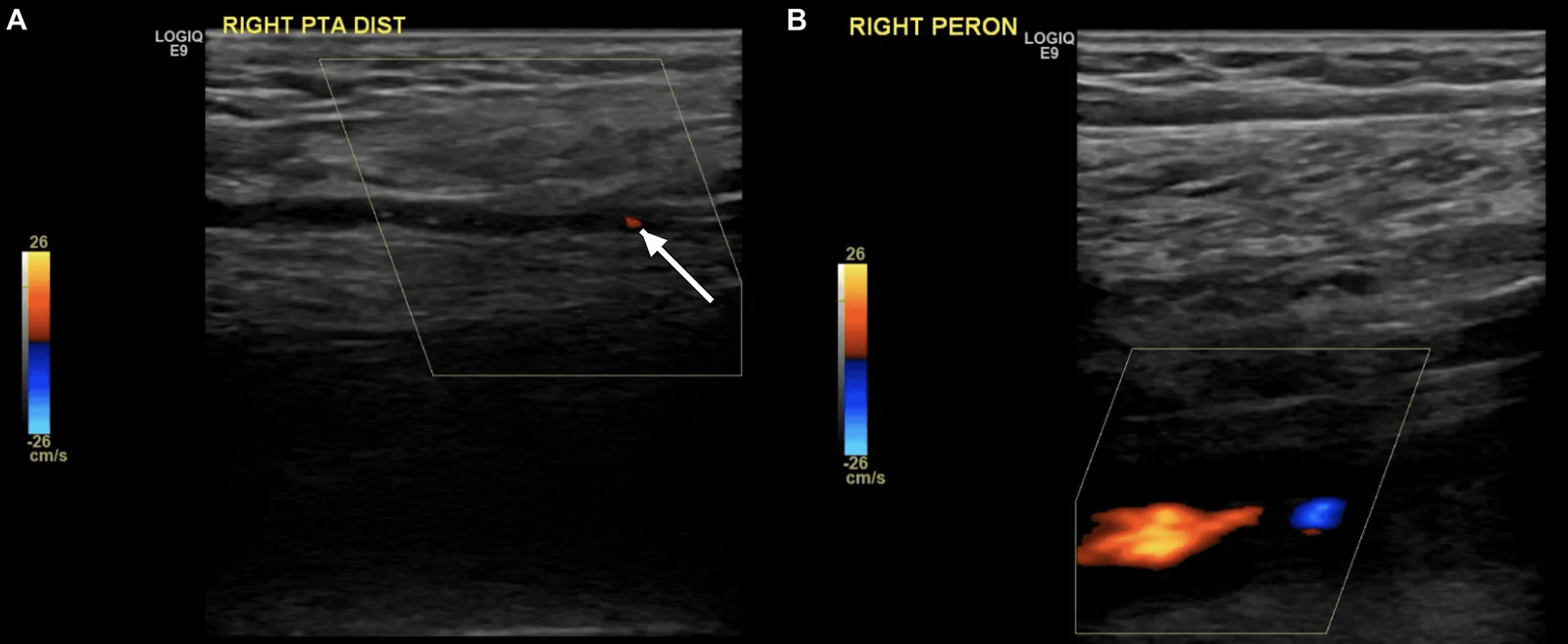
Right lower extremity arterial duplex ultrasonography on admission (A) Distal right posterior tibial artery. Color Doppler demonstrates near-absent luminal flow, with only a punctate focus of residual color signal (arrow), corresponding to the trace flow reported in this segment. (B) Right peroneal artery, shown for comparison. Color Doppler demonstrates normal luminal filling, with the accompanying peroneal vein displayed in blue. The color velocity scale is plus or minus 26 cm/s in both panels. Color Doppler settings differed between segments: panel A, pulse repetition frequency 4.2 kHz and wall filter 767 Hz; panel B, pulse repetition frequency 2.4 kHz and wall filter 439 Hz.

**Figure 2:**
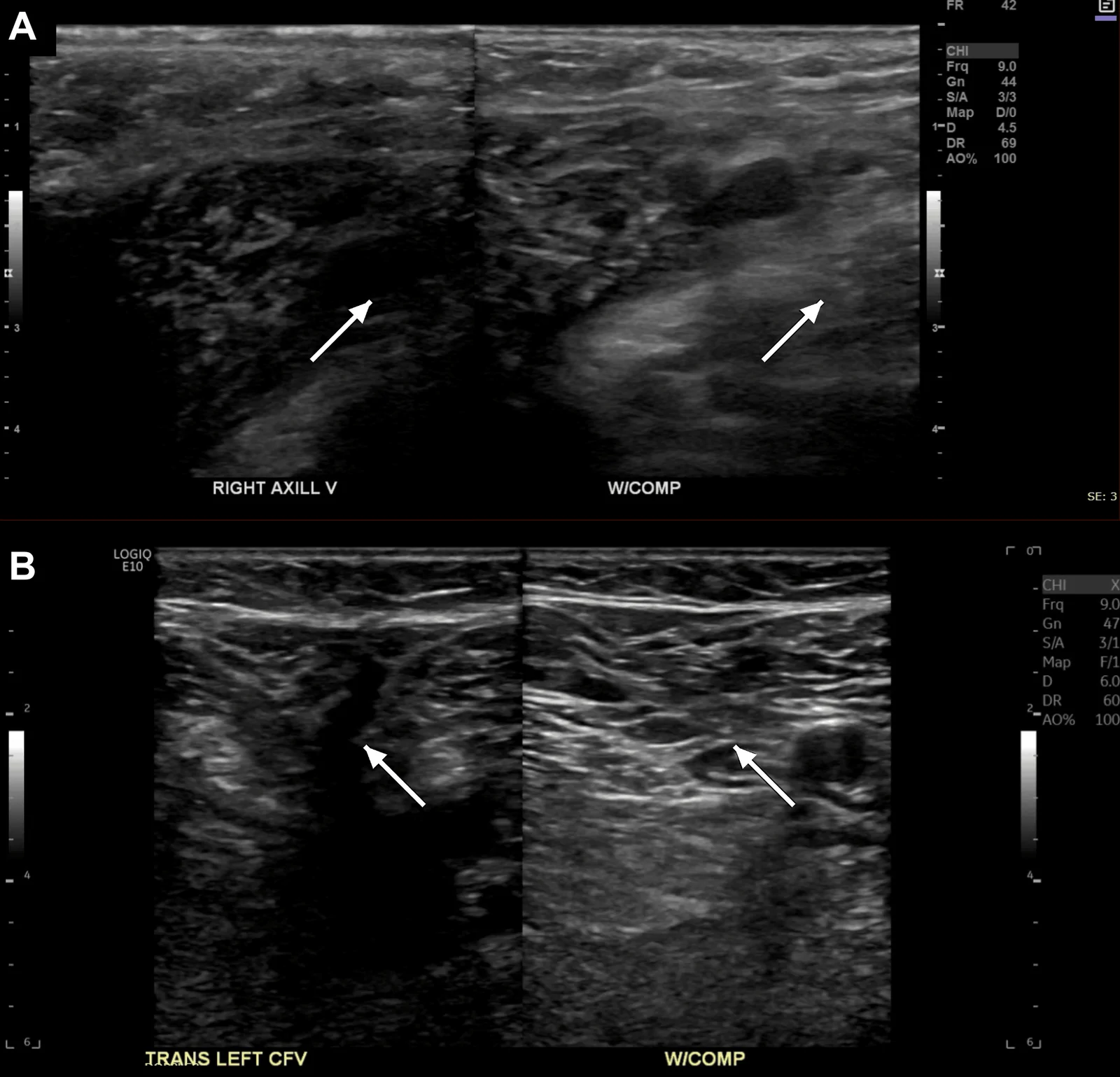
Venous compression ultrasonography of the right upper extremity and the left lower extremity (A) Right axillary vein in transverse section, without (left) and with (right) probe compression. Arrows mark the same venous segment in both images: the lumen is patent in the uncompressed image and is completely apposed on compression. (B) Left common femoral vein in transverse section, without (left) and with (right) probe compression, again arrowed in both images and demonstrating complete collapse. Full compressibility excludes deep venous thrombosis in the interrogated segments.

Between hospital days seven and 15, the patient developed a cholestatic liver injury with resolving pancreatitis - lipase peaking at 1,718 U/L (reference range: 14-79 U/L) on hospital day seven, gamma-glutamyl transferase 958 U/L (reference range: 8-61 U/L), alkaline phosphatase 771 U/L (reference range: 38-126 U/L), and total bilirubin 2.4 mg/dL (reference range: 0.20-1.20 mg/dL) by hospital days 13 to 15, and magnetic resonance cholangiopancreatography on hospital day 14 showing minimal residual peripancreatic edema without biliary obstruction - for which pancreatic enzyme replacement and ursodiol were started.

An arboviral serology panel had been sent on hospital day 11. The ordering rationale was not recorded contemporaneously. At the time it was sent, the patient was in the second week of admission, of Caribbean origin, with bilateral foot pain and swelling not explained by the primary diagnosis or by the limited arterial disease on duplex. The panel was performed at a reference laboratory, and the results were reported on hospital day 15 (Table 2): chikungunya virus immunoglobulin G 3.77 against a reference interval of 0.79 or less negative and 1.10 or greater positive, immunoglobulin M 0.30 (negative), *Chlamydia pneumoniae* immunoglobulin G positive, and Zika and dengue virus serology negative. The problem list that day gained the entry "Chikungunya infection, present on admission", which remained on the list until discharge.

Hospital day 15 was also the first day of recurrent fevers without a clinical focus (a single fever had occurred on hospital day three, at the time of the antibiotic change). Sepsis alerts were recorded on hospital days 15, 17, 18, 23, 24 and 25; the white cell count rose to 22 to 23 × 10^9^/L on hospital days 15 and 16, erythrocyte sedimentation rate exceeded 130 mm/h (reference range: 0.0-20.0 mm/h) and procalcitonin was 1.99 ng/mL on hospital day 15, and lymphocytes fell to 0.85 × 10^9^/L by hospital day 20. Infectious disease consultation was obtained and a fever-of-unknown-origin workup followed: computed tomography of the chest (no pulmonary embolism; prominent subpectoral lymph nodes), magnetic resonance imaging of the brain (age-appropriate), computed tomography and computed tomographic angiography of the abdomen and pelvis (no focus of infection), histoplasma and brucella serologies, QuantiFERON-TB Gold (QIAGEN, Hilden, Germany), malaria smear and antigen, and a venous blood chikungunya virus polymerase chain reaction collected on hospital day 16 and reported as not detected on hospital day 21. That collection fell well outside the viremic window in which chikungunya virus RNA is detectable, so the result was expected to be negative irrespective of prior infection status; the negative immunoglobulin M carries the interpretive weight in this profile. No focus was identified; procalcitonin had normalized by hospital day 25, and the fevers resolved the same day without a diagnosis. 

Differential diagnosis

The bilateral lower extremity symptoms had several competing explanations. What the record documented for and against each is set out here; the weighing of the candidates is reserved for the Discussion.

Dependent or Sepsis-Associated Edema

For: the hand swelling on hospital day seven arose during acute kidney injury with intravenous access in place. Against: swelling and tenderness were documented as present on admission, before fluid resuscitation and before the peak inflammatory burden; the edema was confined to the feet with non-pitting legs; albumin was normal on admission, and there was no proteinuria.

Lumbosacral Radiculopathy or Peripheral Neuropathy

For: raised by the admitting physician on the day of admission; positional, back-originating, radiating pain provoked by standing; paresthesias and weakness on review of systems; pre-admission gabapentin. Against: does not explain the hand swelling. Lumbar spine imaging and electrodiagnostic testing were not performed, so this consideration was never formally evaluated.

Peripheral Arterial Disease

For: trace flow in one right-sided tibial vessel. Against: bilateral symptoms with a normal left leg; palpable pulses, warm feet, no rest pain, no claudication, pain provoked by standing as well as walking; vascular surgery found no acute limb ischemia and recommended conservative management.

Crystalline Arthropathy

For: age, chronic kidney disease, and the small joints of the feet as a characteristic distribution. Against: no gout on the problem list, no prior documented flare, no urate-lowering therapy, no historical uric acid elevation, no joint-focused tenderness, no erythema, no single dominant inflamed joint. Serum uric acid and diagnostic joint aspiration with crystal analysis was not performed, so crystalline arthropathy cannot be formally excluded.

Septic Arthritis

For: confirmed *Klebsiella pneumoniae* bacteremia. Against: bilateral distribution, no dominant or exquisitely tender joint, normal range of motion, no erythema or cellulitis. Joint aspiration was not pursued; in a bacteremic patient, this is a diagnostic limitation.

Post-chikungunya Chronic Arthritis

For: positive immunoglobulin G; origin in a Caribbean country that experienced a sustained chikungunya virus outbreak beginning in 2014 [3]; hand swelling on hospital day seven, hand involvement being characteristic of the syndrome and not explained by a lumbar radicular process. Against: a one-week history against the generally applied criterion of inflammatory polyarthralgia persisting beyond three months from acute infection [4,5,8]; tenderness not focused over any joint; no prolonged morning stiffness documented (the stiffness recorded by physical therapy increased with movement and was accompanied by pain localized to the left knee, the limb with the prior orthopedic procedure); the hand finding is a nursing observation rather than a joint examination, recorded on hospital day seven rather than at presentation, with an unlocated venous study. The available history could not distinguish an acute flare of undocumented chronic post-chikungunya arthropathy from a chronic seropositive state unrelated to the presentation.

Post-infectious Arthropathy Attributed to Chlamydia pneumoniae

For: positive immunoglobulin G. Against: no respiratory illness; a single positive immunoglobulin G in a patient in his 70s does not establish active or recent infection; no titer, no paired sera; and the evidence linking *Chlamydia pneumoniae* to reactive arthritis is weaker than that for *Chlamydia trachomatis* [9].

Recurrent Fever

This was considered separately from the foot pain. Blood cultures were negative from hospital day five; there was no focus on cross-sectional imaging; broad serology was negative; the fever resolved by hospital day 25. No unifying diagnosis for the fevers was documented.

Treatment

Empirical ceftriaxone was initiated as part of the sepsis bundle on admission and changed to meropenem on hospital day three, when fever occurred and an extended-spectrum beta-lactamase phenotype was suspected - 1 g intravenously every 12 hours at an estimated glomerular filtration rate of 35 mL/min/1.73 m^2^, increased to every eight hours as renal function recovered, for a planned 14-day course. Repeat blood cultures were negative from hospital day five onward. During the febrile period, the patient also received empirical vancomycin and micafungin; micafungin was restarted on hospital day 15, and vancomycin was again in use on hospital day 26. The indications were not stated in the record, and no fungal or gram-positive target was identified. Repeat and surveillance blood cultures are not routinely indicated after gram-negative bacteremia; the hospital day 24 set was drawn during the febrile period on a day a sepsis alert was recorded, and no further rationale was documented.

Acute chikungunya virus infection more commonly produces leukopenia and lymphopenia than leukocytosis [10]; the admission leukocytosis recorded here was attributable to the bacteremia and was not interpreted as evidence bearing on arboviral activity.

Analgesia was constrained on three sides: non-steroidal anti-inflammatory drugs (NSAIDs) were withheld with the documented notation "no NSAIDs due to kidney disease", acetaminophen was discontinued on hospital day 15 for the cholestatic liver injury, and tramadol and morphine were used as needed. Corticosteroids were started in the fourth week of admission, between hospital days 21 and 24, after the fever workup had returned negative and blood cultures had been negative since hospital day five, for the arthropathy as characterized by the clinical team: intravenous methylprednisolone 40 mg every 24 hours, transitioned to oral prednisone 20 mg, with a taper planned at discharge although a detailed schedule was not specified. The severity of the arthropathy was not formally graded: no tender or swollen joint count or other structured measure was recorded. Its functional impact is documented only indirectly - difficulty walking because of pain from presentation, as-needed opioid analgesia, and inpatient physical therapy; the discharge to a skilled nursing facility reflects the hospitalization as a whole rather than the arthropathy alone. Doxycycline 100 mg orally twice daily first appears on the medication list on hospital day 17, the morning after infectious diseases recommendations were recorded, and was continued for at least 10 days and completed before discharge. The notes record no indication for it. The *Chlamydia pneumoniae* immunoglobulin G is the only documented finding the course corresponds to, and the discharge summary later attributed the arthropathy to that organism; a single positive immunoglobulin G does not, however, support an empiric course of doxycycline, and the course is reported as given rather than as recommended practice. Physical medicine and rehabilitation was consulted and inpatient physical therapy was initiated; rheumatology evaluation at an academic center was recommended for after discharge.

Outcome and follow-up

After a hospitalization of 29 days, attributed by the discharge documentation to recurrent fevers, elevated liver enzymes, and acute kidney injury, the patient was assessed by inpatient rehabilitation, did not qualify, and was discharged to a skilled nursing facility for continued therapy. Discharge medications included prednisone 20 mg orally, metoprolol, doxazosin, empagliflozin (started for newly identified diabetes), oral sodium bicarbonate, gabapentin, and a probiotic; non-steroidal anti-inflammatory drugs were not prescribed. Outpatient referrals were placed to family practice, nephrology, and hematology-oncology, with follow-up arranged through an advanced-practice provider. The hospitalist who took over care on hospital day 21 (JHF) recorded slow clinical improvement on hospital day 23 and, on hospital day 24, "likely developed chronic inflammatory polyarthritis s/p previous Chikungunya infection", citing by title a 36-month prospective cohort of 180 viremic patients from the 2006 La Réunion Island outbreak, 60% of whom experienced arthralgia during follow-up [4], and noting that a negative polymerase chain reaction does not exclude prior infection. The discharge summary three days later, written by the attending physician of record, attributed the polyarthritis to prior *Chlamydia pneumoniae* infection. No post-discharge follow-up data were available in the records reviewed; the corticosteroid response, any subsequent rheumatologic evaluation, and the patient's longer-term functional status could therefore not be ascertained.

## Discussion

This case illustrates a clinical-decision question that is poorly addressed in the published case literature: when an inpatient who already carries a primary acute diagnosis has an unexplained musculoskeletal complaint, what can arboviral serology decide, and what happens to a predictable positive result once it is in the chart?

The serologic profile encountered here - chikungunya virus immunoglobulin G positive with both immunoglobulin M and polymerase chain reaction negative - is the expected signature of prior, rather than active, infection [1,2]. It cannot establish a temporal link between prior infection and current symptoms; it cannot distinguish symptomatic from subclinical prior infection; and it cannot confirm post-chikungunya chronic arthritis, which requires inflammatory polyarthralgia persisting beyond three months from acute infection with the characteristic small-joint, symmetric, polyarticular distribution and prolonged morning stiffness [4,5,8]. The one-week history in this patient failed the temporal criterion; neither joint-focused tenderness nor prolonged morning stiffness was documented; and the single finding pointing beyond the feet - hand swelling on hospital day seven - is a nursing observation with an untested thrombotic alternative. The case does not establish post-chikungunya chronic arthritis, and the authors do not claim it. Yet the result entered the problem list on the day it returned as "Chikungunya infection" and remained there for 11 days: the reference laboratory's own comment, "suggests current or past infection", did not prevent the entry.

The two attributions that the treating physicians went on to make rest on the same class of evidence: a single untitered immunoglobulin G in a man in his 70s, without paired sera and without any capacity to establish timing. Both results were probable before the blood was drawn. Chikungunya seroprevalence in the post-2014 Caribbean is substantial [1-3], and *Chlamydia pneumoniae* immunoglobulin G seropositivity reached 98.6% above age 70 in a 12,050-specimen series [11]. The two attributions are nonetheless not equally supported as explanations. The association between chikungunya virus and a chronic inflammatory polyarthralgia of the small joints of the hands and feet is established in prospective cohorts followed to 32 and 36 months [4,6] and in a cross-sectional survey more than two years after infection [5], and specific management guidance exists [8]; the evidence linking *Chlamydia pneumoniae* to reactive arthritis is substantially weaker, reactive arthritis being best characterized following *Chlamydia trachomatis* [9]. The hand swelling, if inflammatory, fits the former and not the latter. So if a post-infectious label was to be applied on serology alone, chikungunya was the less unsupported of the two - which is the most that can be said, and it is not a diagnosis. Two attributions were in fact documented three days apart: the hospitalist who took over care in the fourth week recorded a probable post-chikungunya arthropathy on hospital day 24, citing the cohort literature and noting that a negative polymerase chain reaction does not exclude prior infection, and the discharge summary named *Chlamydia pneumoniae*. That two clinicians reading the same results reached different attributions is the point: the results could not adjudicate between them.

Three readings of one panel

The two positive immunoglobulin G results were read three ways in the course of one admission. On the day they returned, the chikungunya result was entered on the problem list as an active infection and managed with supportive care. Over the following week the *Chlamydia pneumoniae* result was treated as an indication for antibiotics. On hospital day 24, after that week had passed with slow clinical improvement and the fever workup had returned negative, the hospitalist who had taken over care read the chikungunya result as what the serologic profile indicates - prior infection - and proposed it as the explanation for a chronic inflammatory polyarthritis, citing the supporting cohort literature. Only the third reading is consistent with the serology, and even it is a framing rather than a diagnosis, because the temporal criterion and the joint pattern were not documented. The sequence shows what a predictable positive result does once it is in the chart, and how much depends on who reads it and when.

Should the panel have been sent? It is fair to weigh this. Against: acute chikungunya was never plausible - no fever before admission, no rash, symptoms of one week, no documented recent travel - so the immunoglobulin M could not reasonably have been positive, and a positive immunoglobulin G was predictable for this patient and could not settle the attribution on its own. For: in a patient from an endemic region with an unexplained inflammatory polyarthralgia and, from hospital day 15, unexplained fever, arboviral serology is a conventional component of the workup, and the result supplied the working framework within which the arthropathy was eventually characterized and rheumatologic follow-up arranged. What would have sharpened the decision either way is the history the record lacks: the recency of potential exposure, the incubation interval, the onset of the joint symptoms relative to any prior febrile illness, and the presence or absence of acute arboviral features. With those established, the clinician can say before ordering which result would change the plan; without them, a positive immunoglobulin G is more likely to be ordered than to be informative.

Treating without a diagnosis is the other half of the case. With non-steroidal anti-inflammatory drugs contraindicated by chronic kidney disease, acetaminophen withdrawn for cholestatic liver injury, a patient in persistent pain on opioids, and cultures and cross-sectional imaging clear, the empirical corticosteroid trial is defensible as symptom management regardless of which label was attached to the arthropathy; management guidance for post-chikungunya rheumatic disorders supports corticosteroids when rheumatologic evaluation is delayed [8]. Doxycycline was started empirically on the *Chlamydia pneumoniae* result during the febrile period, with infectious diseases consulted and no focus identified. In retrospect a single immunoglobulin G does not establish active infection, and the course is the second of the three readings described above. The empirical vancomycin and micafungin courses and the repeat blood cultures reflect the same uncertainty during three weeks of unexplained fever.

Several limitations constrain the conclusions of this case. No structured joint examination was performed at any point in the hospitalization; the examination findings reported here were assembled from the admission history and physical examination, the vascular surgical consultation, and nursing assessments, none of which constituted a dedicated musculoskeletal evaluation. Lumbar spine imaging and electrodiagnostic testing were not performed, so the radicular explanation was never formally evaluated. Rheumatologic consultation was not obtained during the admission; diagnostic joint aspiration and serum uric acid measurement were not performed, and rheumatoid factor and anti-citrullinated peptide antibody were not tested, although erythrocyte sedimentation rate and procalcitonin were and are reported. The results of the left upper extremity venous study and of the echocardiogram ordered on hospital day four were not located in the records reviewed, so the single finding favoring an inflammatory process beyond the feet carries an untested thrombotic competing explanation, and endocarditis was not documented as excluded in a bacteremic patient with recurrent fever. The migration and travel history was not documented, so the timing of any prior chikungunya virus infection could not be established. The recurrent fevers were never explained. The reasoning that prompted the arboviral panel was not recorded contemporaneously, and none of the authors was involved in the patient's care at the time it was sent, so no ordering rationale is presented. The two student authors did not participate in the patient's care. The two color Doppler acquisitions shown in Figure 1 were obtained with different pulse repetition frequency and wall filter settings; because higher settings preferentially suppress low-velocity flow, the near-absent color signal in the posterior tibial artery is best interpreted alongside the formal vascular laboratory report rather than as an independent quantification of stenosis severity. Finally, no post-discharge follow-up data were available, so the response to corticosteroids and the longer-term course of the arthropathy could not be assessed.

## Conclusions

A positive chikungunya virus immunoglobulin G is common in adults from the Caribbean and, taken alone, indicates prior exposure; a positive *Chlamydia pneumoniae* immunoglobulin G is near-universal in a man in his 70s. Neither dates an infection, ties it to the presenting complaint, or establishes a post-infectious arthropathy, and in this patient - foot pain of one week, no documented temporal criterion, no documented characteristic joint pattern - neither did. Two physicians attributed the same findings to different organisms on the same evidence, which is the clearest demonstration that the evidence could not decide. The chikungunya result was carried on the problem list as an active infection for 11 days, and the reading consistent with the serology - prior infection - was recorded on hospital day 24, nine days after the result returned and a week into an antibiotic course for the companion result.

The transferable lesson is about reading the result rather than ordering the test. A single positive immunoglobulin G, whichever organism it names, can be read three ways. Read as an active infection, it misdirects the problem list; read as an indication for treatment, it misdirects therapy; read as prior exposure, it becomes a framing tool that sends the clinician back to the history, the joint examination, and the competing diagnoses - lumbosacral radiculopathy when pain is positional or radiates from the spine, crystalline arthropathy in older patients with chronic kidney disease, and septic arthritis in the setting of bacteremia. Establishing the recency of exposure, the incubation interval, and the onset of joint symptoms before the panel is sent tells the clinician whether any result would change the plan and allows a positive result to be read as prior exposure on the day it returns rather than nine days later. Where the diagnosis remains uncertain after history, examination, and serology, this case supports joint aspiration, where an effusion permits, and rheumatologic evaluation before any commitment to long-term immunomodulatory therapy.
